# Long-term cardiovascular risk of hypertensive events in emergency department: A population-based 10-year follow-up study

**DOI:** 10.1371/journal.pone.0191738

**Published:** 2018-02-15

**Authors:** Sihyoung Lee, Chang-youn You, Joonghee Kim, You Hwan Jo, Young Sun Ro, Si-Hyuck Kang, Heeyoung Lee

**Affiliations:** 1 Department of Emergency Medicine, Seoul National University Bundang Hospital, Bundang-gu, Seongnam-si, Gyeonggi-do, Republic of Korea; 2 Laboratory of Emergency Medical Services, Seoul National University Hospital Biomedical Research Institute, Jongno-gu, Seoul, Republic of Korea; 3 Department of Cardiology, Cardiovascular Center, Seoul National University Bundang Hospital, Bundang-gu, Seongnam-si, Gyeonggi-do, Republic of Korea; 4 Department of Preventive Medicine, Seoul National University Bundang Hospital, Bundang-gu, Seongnam-si, Gyeonggi-do, Republic of Korea; Azienda Ospedaliera Universitaria di Perugia, ITALY

## Abstract

**Background:**

Hypertension-related visits to the emergency department (ED) are increasing every year. Thus, ED could play a significant role in detecting hypertension and providing necessary interventions. However, it is not known whether a hypertensive event observed in the ED is an independent risk factor for future major adverse cardiovascular events (MACE).

**Methods:**

A population-based observational study was conducted using a nationally representative cohort that contained the claim data of 1 million individuals from 2002 to 2013. We included non-critical ED visits without any history of MACE, and compared the new occurrences of MACE according to the presence of hypertensive events using extended Cox regression model. The disease-modifying effect of a follow-up visit was assessed by analyzing the interaction between hypertensive event and follow-up visit.

**Results:**

Among 262,927 first non-critical ED visits during the study period (from 2004 to 2013), 6,243 (2.4%) visits were accompanied by a hypertensive event. The hypertensive event group had a higher risk of having a first MACE at 3 pre-specified intervals: 0–3 years (HR, 4.25; 95% CI, 3.83–4.71; P<0.001), 4–6 years (HR, 3.65; 95% CI, 3.14–4.24; P<0.001), and 7–10 years (HR, 3.20; 95% CI, 2.50–4.11; P<0.001). Follow-up visits showed significant disease-modifying effect at 2 intervals: 0–3 years (HR 0.65, 95% CI, 0.50–0.83) and 4–7 years (HR 0.68, 95% CI, 0.48–0.95).

**Conclusions:**

A hypertensive event in the ED is an independent risk factor for MACE, and follow-up visits after the event can significantly modify the risk.

## Introduction

Hypertension is one of the most important risk factors for cardiovascular diseases. It is modifiable and numerous studies proved the beneficial effects of blood pressure control [1–4]. Because hypertension is usually asymptomatic, patients often do not seek care until significant damages have occurred making effective control challenging.

It has been shown that hypertension is common in emergency departments (EDs), hence EDs might have a role as a potential screening site for detecting hypertension [5–9]. It has thus been suggested that nationwide interventions should take place to help EDs take a proactive role in ensuring that patients with uncontrolled hypertension get continued care after their visits. However, there are some knowledge gaps that should be filled before implementing such interventions. First, it is unknown whether hypertension observed in the ED is an independent risk factor for future major adverse cardiovascular events (MACE). Second, it is also unknown whether subsequent ambulatory visits can make any significant impact on long-term outcomes.

We conducted a population-based observational study using a nationally representative cohort to fill the knowledge gaps. The primary objective was to determine whether hypertension in the ED is a significant risk factor for the development of MACE. The secondary objective was to determine whether follow-up visits can modify the increased risk in a favorable way.

## Materials and methods

### Data source

The data source was the National Health Insurance Service-National Sample Cohort (NHIS-NSC), a population-based cohort established by the Korean NHIS [10]. It contains de-identified claim information of 1 million individuals who were randomly sampled after stratification from the entire Korean population. It provides diagnostic codes based on the International Classification of Diseases (ICD)-10 coding system, prescription and procedure codes, and related costs, as well as demographic information such as age, sex, and socioeconomic status. It also has information about disability and death based on the national disability registration data and death certificates, respectively. We used the most recent release, which contains claim data from 2002 to 2013. The detailed descriptions of the cohort data can be found in a previous paper [10]. Seoul National University Bundang Hospital (SNUBH) institutional review board (IRB) approved the analysis and provided a consent waiver.

### Case selection and data handling

We included ED visit cases of adult (≥20 years old) patients from January 2004 to September 2013 for non-critical conditions (index visit). The non-criticality was defined as discharge from hospital within the same day or on the next day without any subsequent admission within a week. We set two years of washout period prior to the index visit and excluded cases with any entry of diagnostic codes for acute coronary syndrome (ACS), heart failure or stroke as well as any procedure codes for revascularization and pacemaker insertion during the two years as well as at the index visit. If a patient had multiple ED visits fulfilling these criteria, only the first event was used for analysis.

The main exposure, which was the ED hypertensive event was defined based on the action of the physicians, which included any of the following: 1) entry of any ICD code for hypertension (I10.x, I11.x, I12.x, I13.x, I15.x and R03.0x); 2) administration of any of the following intravenous (IV) antihypertensive agents during the index visit: labetalol (Anatomical Therapeutic Chemical [ATC] code, C07AG01), nicardipine (ATC code, C08CA04), hydralazine (ATC code, C02DB02), or nitroprusside (ATC code, C02DD01), and/or; 3) prescription of any of the oral antihypertensive agents as defined in a previous study [11]. Another exposure, which was a follow-up visit, was defined as an ambulatory visit with any of the ICD codes for hypertension (I10.x, I11.x, I12.x, I13.x, I15.x and R03.0x) or prescription of any of the oral antihypertensive agents for a week or more during the first 3 months after the index visit [11]. Cases with censoring event (end of cohort observation, e.g. December, 31, 2013 or death) during the 3-month window were excluded.

From the end of the 3-month period, patients were observed for a new occurrence of a MACE, which includes ACS, revascularization, acute stroke, decompensated heart failure, pacemaker insertion and cardiovascular death until death or December 31, 2013. To determine the presence of each comorbidity, including hypertension, diabetes mellitus, dyslipidemia, atrial fibrillation/flutter, ischemic heart disease, peripheral artery disease, chronic renal failure, end-stage renal disease, advanced liver disease, chronic obstructive pulmonary disease (COPD) and malignancy, data from the past 2 years prior to the index visit were scanned, based on the criteria determined a priori. The study period was from January 2004 to September 2013, because the preceding 2 years were required to determine comorbidities while the following 3 months were needed to determine the occurrence of the follow-up visit. The detailed description of the codes used for definitions of outcome events and comorbidities are available in S1 and S2 Tables in S1 File.

### Statistical analysis

Categorical variables were reported using frequencies and proportions, while continuous variables were reported using medians and interquartile ranges (IQRs). Wilcoxon’s rank-sum test, the chi-square test, or Fisher’s exact test was performed as appropriate, for comparisons between groups.

We used an extended Cox model using time-varying coefficients (TVC) to determine the subsequent risk of MACE after the ED hypertensive event [12]. Specifically, we estimated the hazard ratios (HRs) and their 95% confidence intervals (CIs) for MACE at 3 pre-specified intervals after the index ED visit (0–3 years, 4–6 years, and 7–10 years), since the clinical significance of either the ED hypertensive event or the follow-up visit could change during the 10-year observation period. Using the method, we first obtained the crude risk of having a MACE for each of the 3 intervals. Then, we assessed the disease-modifying effect of a follow-up visit by incorporating an interaction term between the hypertensive event and the follow-up visit. The interaction was examined both with and without adjustment for other covariates, including age, sex, comorbidities, and household income levels, categorized as low, mid, and high. The goodness of fit of the multivariable models was tested with Gronnesby and Borgan’s goodness-of-fit test and their proportional hazard assumption was assessed by examination of Schoenfeld residual plots. The results of the Cox regression analyses were presented as hazard ratios (HRs), and their 95% confidence intervals (CIs) were also presented.

*P*-values < 0.05 were considered significant. All data handling and statistical analyses were performed using R-packages version 3.3.2 (R Foundation for Statistical Computing, Vienna, Austria).

## Results

**Fig 1:**
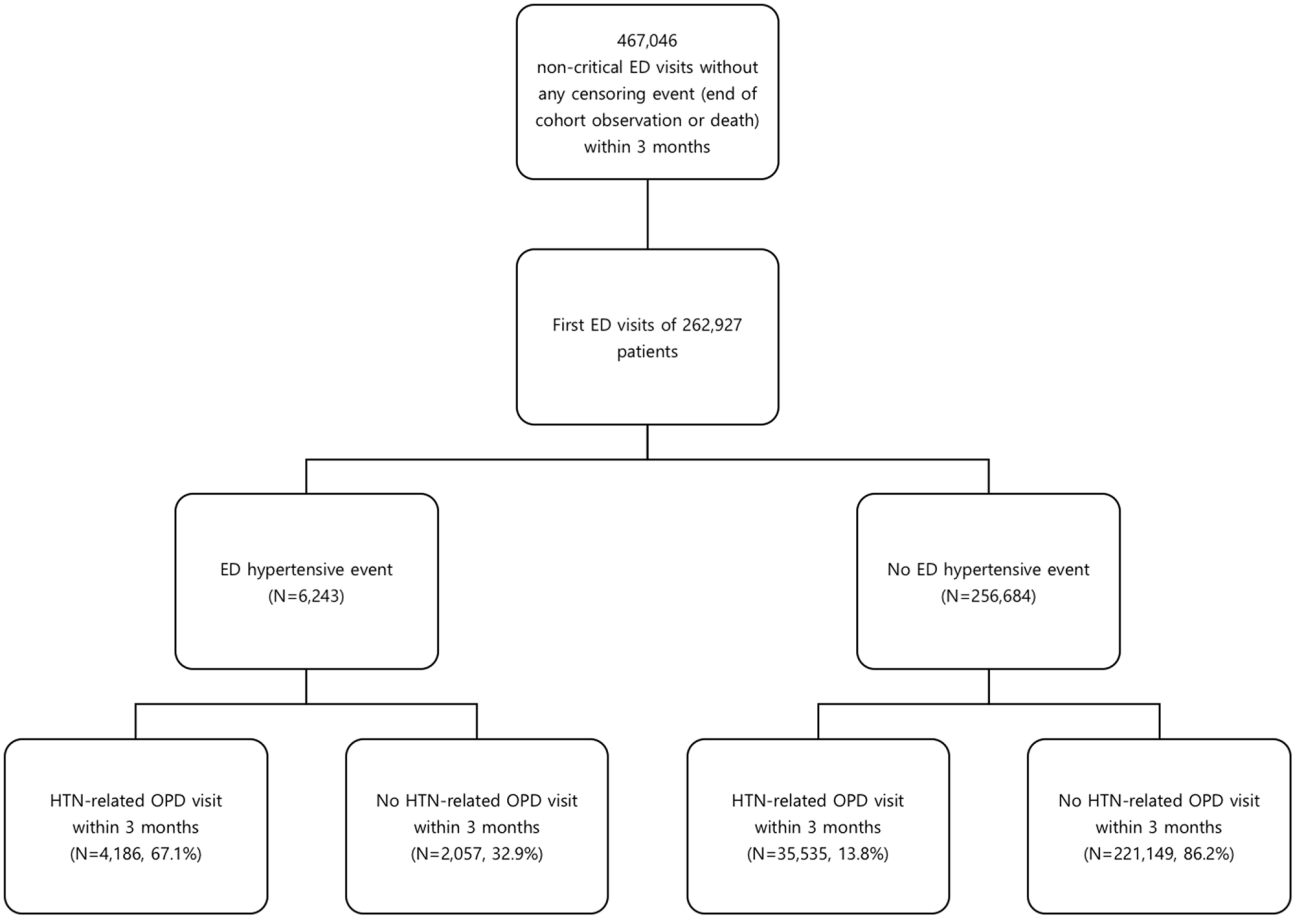
Flow diagram of case selection and group allocation. (ED, emergency department; HTN, hypertension; OPD, out-patient department).

Fig 1 shows the flow diagram of case selection and group allocation (Fig 1). Among the 467,046 non-critical ED visits, 262,927 first visits were included in the study. Among the cases, a total of 6,243 (2.4%) visits were identified as being accompanied by a hypertensive event (Table 1). Hypertensive events were associated with age (p<0.001), sex (p<0.001), income class (p<0.001), and every comorbidity assessed (all p<0.001). Outpatient visits for hypertension within 3 months were identified in 4,186 (67.1%) in the hypertensive event group and in 35,535 (13.8%) in the no hypertensive event group.

**Table 1:** Baseline characteristics of study population.

	Hypertensive event (N = 6243)	No event (N = 256684)	*p*
**Age**	57.0 (IQR, 47.0–69.0)	41.0 (IQR, 30.0–52.0)	<0.001
**Sex**			<0.001
Male	2964 (47.5%)	128933 (50.2%)	
Female	3279 (52.5%)	127751 (49.8%)	
**Household income level**			<0.001
High (8-10th decile)	1580 (25.3%)	59987 (23.4%)	<0.001
Middle (4-7th decile)	2110 (33.8%)	101390 (39.5%)	<0.001
Low (1-3th decile and medical assistance beneficiary)	2553 (40.9%)	95307 (37.1%)	<0.001
**Comorbidities**			
Hypertension	3440 (55.1%)	33513 (13.1%)	<0.001
Diabetes mellitus	957 (15.3%)	12344 (4.8%)	<0.001
Dyslipidemia	1567 (25.1%)	25256 (9.8%)	<0.001
Atrial fibrillation/flutter	82 (1.3%)	762 (0.3%)	
Ischemic heart disease	590 (9.5%)	6001 (2.3%)	<0.001
Peripheral arterial disease	701 (11.2%)	9157 (3.6%)	<0.001
Chronic renal failure	179 (2.9%)	1071 (0.4%)	<0.001
End-stage renal disease	88 (1.4%)	334 (0.1%)	<0.001
Advanced liver disease	162 (2.6%)	1951 (0.8%)	<0.001
Chronic obstructive pulmonary disease	207 (3.3%)	3146 (1.2%)	<0.001
Malignancy	318 (5.1%)	7644 (3.0%)	<0.001
**Outpatient visit for hypertension within 3 months**	4186 (67.1%)	35535 (13.8%)	<0.001
**Occurrence of outcome events**			
Major cardiovascular event	643 (10.3%)	6853 (2.7%)	<0.001
Acute coronary syndrome	129 (2.1%)	1430 (0.6%)	<0.001
Revascularization	104 (1.7%)	1177 (0.5%)	<0.001
Stroke	349 (5.6%)	4125 (1.6%)	<0.001
Admission for heart failure	108 (1.7%)	823 (0.3%)	<0.001
Pacemaker application	18 (0.3%)	125 (0.0%)	<0.001
Cardiovascular death	118 (1.9%)	1053 (0.4%)	<0.001

IQR, interquartile range

**Fig 2:**
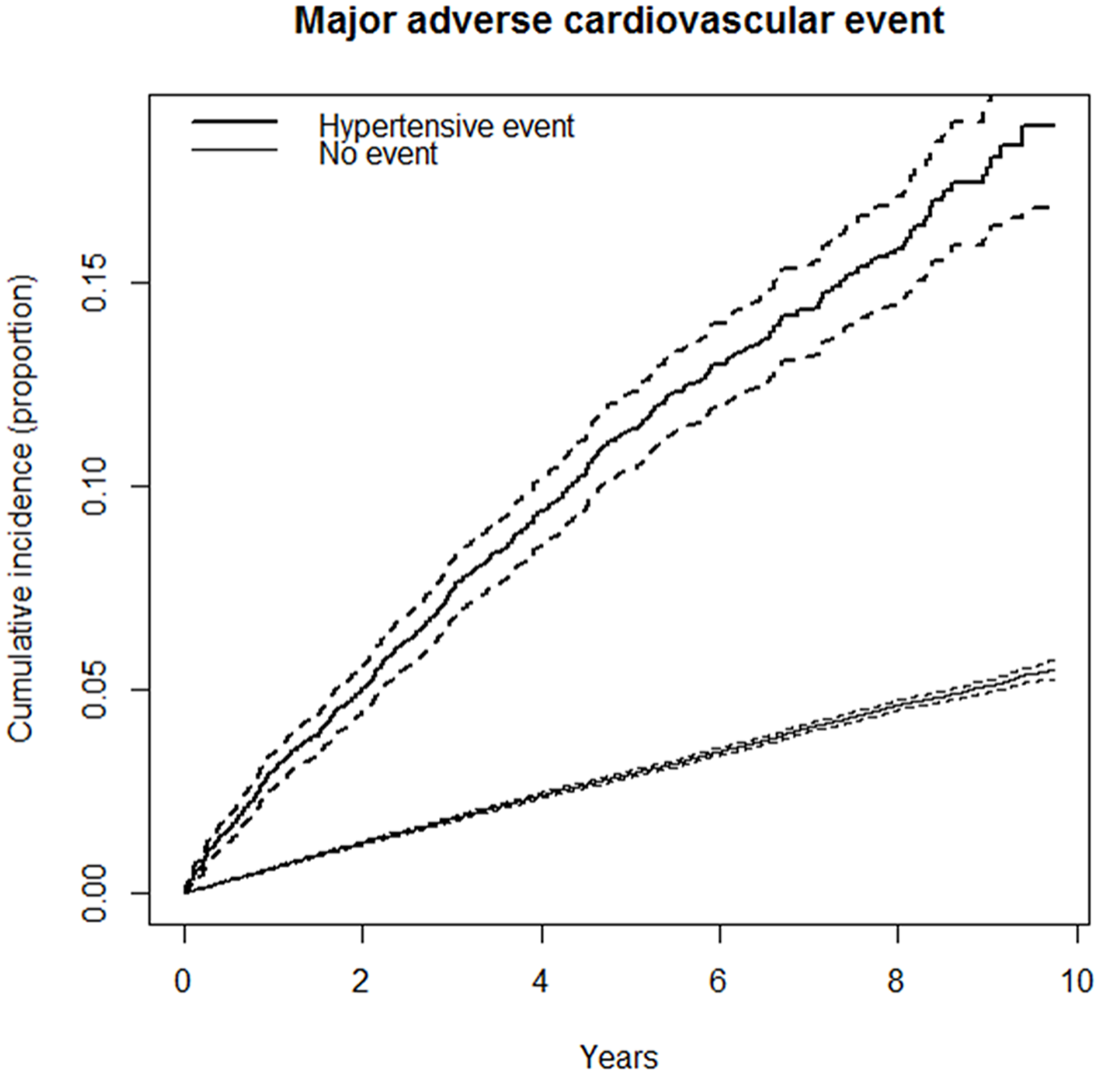
Cumulative incidence of total major adverse cardiovascular event (MACE) according to the presence of hypertensive event in ED.

**Fig 3:**
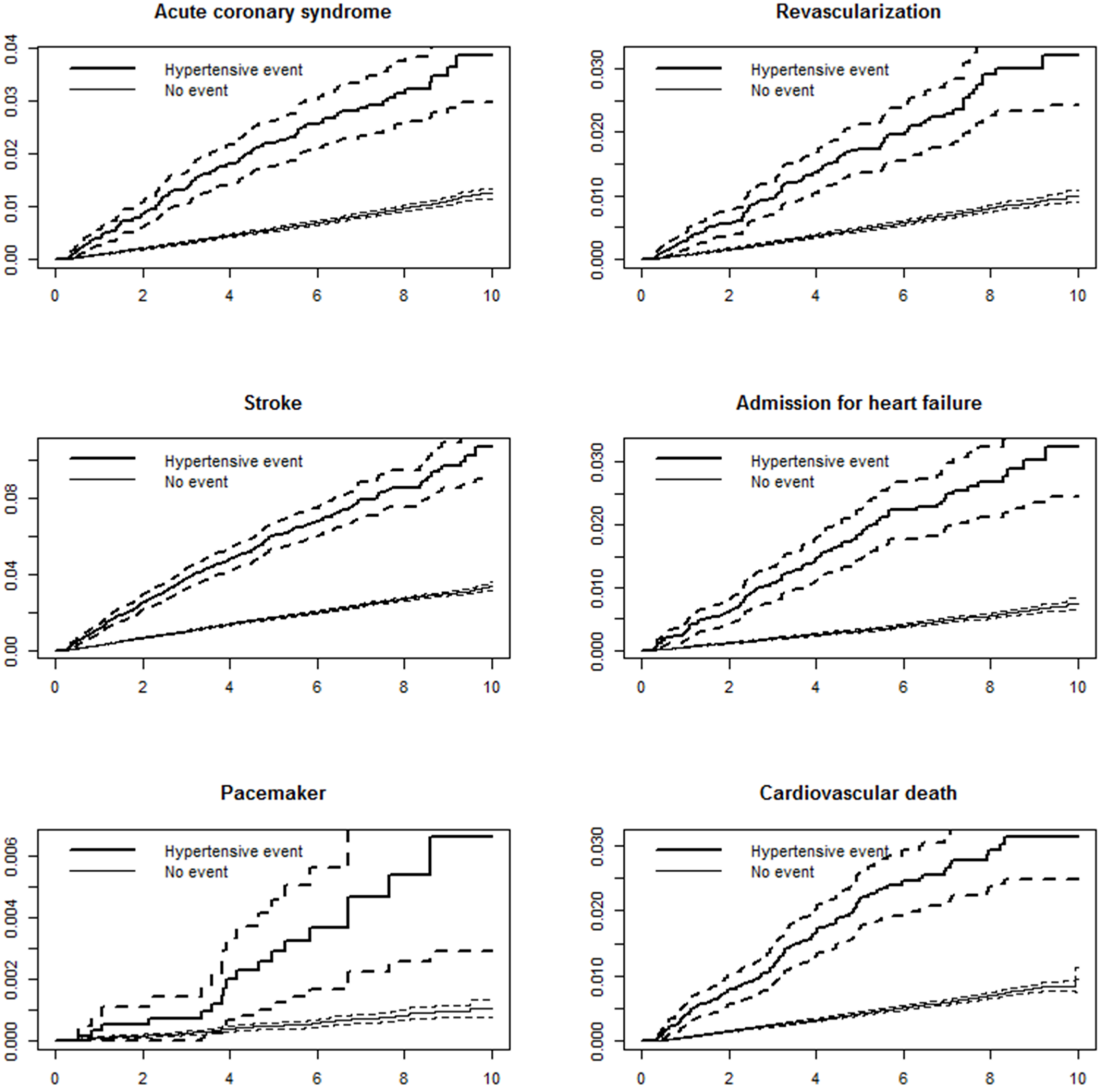
Cumulative incidence of each components of major adverse cardiovascular event (MACE) according to the presence of hypertensive event in ED. x axis: years, y axis: cumulative incidence described in proportion.

Using the extended Cox model with TVC, we first obtained the crude risk of having a first MACE at 3 pre-specified intervals: 0–3 years (HR, 4.25; 95% CI, 3.83–4.71; *P*<0.001), 4–6 years (HR, 3.65; 95% CI, 3.14–4.24; *P*<0.001), and 7–10 years (HR, 3.20; 95% CI, 2.50–4.11; *P*<0.001). Figs 2 and 3 show the cumulative incidence of MACE and its components with and without an ED hypertensive event.

We modelled the interaction between hypertensive events and follow-up visits. The disease-modifying effect of a follow-up visit was significant throughout the study period, with HRs of 0.31 (95% CI, 0.25–0.40), 0.34 (95% CI, 0.24–0.48), and 0.50 (95% CI, 0.29–0.89), for each of the intervals, respectively (Table 2). When adjusted for covariates, including age, sex, hypertension, diabetes mellitus, dyslipidemia, atrial fibrillation/flutter, ischemic heart disease, peripheral artery disease, chronic renal failure, end-stage renal disease, advanced liver disease, COPD, malignancy, and household income levels, the disease-modifying effect was still significant up to the second interval (0–6 years), with HRs of 0.65 (95% CI, 0.50–0.83), 0.68 (95% CI, 0.48–0.95), and 0.99 (95% CI, 0.56–1.75), respectively (Tables 2 and 3).

**Table 2:** Effect size of ED hypertensive event with and without follow-up visit.

	Unadjusted			Adjusted		
	0–3 years	4–6 years	7–10 years	0–3 years	4–6 years	7–10 years
Without following visit	4.51 (3.65–5.58)	3.64 (2.72–4.86)	2.61 (1.61–4.23)	1.99 (1.61–2.47)	1.73 (1.29–2.32)	1.27 (0.78–2.05)
With following visit	1.42 (1.25–1.60)	1.25 (1.04–1.50)	1.32 (0.97–1.79)	1.29 (1.14–1.46)	1.17 (0.98–1.40)	1.25 (0.93–1.70)
Difference in difference	0.31 (0.25–0.40)	0.34 (0.24–0.48)	0.50 (0.29–0.89)	0.65 (0.50–0.83)	0.68 (0.48–0.95)	0.99 (0.56–1.75)

**Table 3:** Multivariable Cox-regression model for major adverse cardiovascular event.

	Hazard ratio (95% confidence interval)	*P*
Age, years, per 1 year	1.07 (1.07–1.07)	<0.001
Sex, female	0.63 (0.60–0.66)	<0.001
Hypertension	1.34 (1.24–1.45)	<0.001
Diabetes mellitus	1.38 (1.29–1.47)	<0.001
Dyslipidemia	1.03 (0.97–1.09)	<0.001
Atrial fibrillation or flutter	1.67 (1.39–2.01)	0.965
Ischemic heart disease	1.41 (1.30–1.53)	<0.001
Peripheral arterial disease	1.00 (0.92–1.08)	0.386
Chronic renal failure	1.55 (1.26–1.90)	<0.001
End-stage renal disease	2.06 (1.51–2.82)	<0.001
Advanced liver disease	1.25 (1.05–1.49)	0.013
Chronic obstructive pulmonary disease	1.05 (0.94–1.18)	0.372
Malignancy	0.89 (0.81–0.99)	0.036
High-income level (8–10 decile)	0.92 (0.87–0.97)	0.002
Mid-income level (4–7 decile)	1.06 (1.00–1.13)	0.056
Low-income level (1–3 decile and medical assistance program beneficiary)	Baseline	
ED hypertensive event (0–3 years)	1.99 (1.61–2.47)	<0.001
ED hypertensive event (4–6 years)	1.73 (1.29–2.33)	<0.001
ED hypertensive event (7–10 years)	1.27 (0.78–2.05)	0.336
Hypertension-related ambulatory visit (0–3 years)	1.27 (1.17–1.39)	<0.001
Hypertension-related ambulatory visit (4–6 years)	1.23 (1.10–1.36)	<0.001
Hypertension-related ambulatory visit (7–10 years)	1.02 (0.87–1.20)	0.770
Interaction between hypertensive event and following visit (0–3 years)	0.65 (0.50–0.83)	<0.001
Interaction between hypertensive event and following visit (4–6 years)	0.68 (0.48–0.95)	0.026
Interaction between hypertensive event and following visit (7–10 years)	0.99 (0.56–1.75)	0.972

## Discussion

In this population-based longitudinal study, we assessed whether a hypertensive event in the ED is a risk factor for MACE. We found that it was associated with a significantly increased risk of MACE and each of its components. We also found that the effect size of the hypertensive event was significantly different according to the presence of a follow-up visit within 3 months. To our knowledge, these are novel findings and suggest the importance of appropriate follow-up and continued care for hypertension in the ED.

Although it has been reported that hypertension-related ED visits are increasing [8,13], there have been few scientific reports on the risk of long-term cardiovascular complications in patients with hypertensive events in the ED. Frei *et al*. [14] revealed that ED patients (n = 149) with hypertension or a chief complaint of high blood pressure had low risk of serious outcomes within 7 days. Levy *et al*. [15] demonstrated that acute blood pressure management in ED was not associated with revisits up to 30 days and mortality up to a year in patients (n = 1,016) with markedly elevated blood pressures without acute target organ damage (TOD). In a retrospective, multi-center, cohort-crossover study of patients discharged from the ED with a primary diagnosis of hypertension (n = 552,569), the risk of having an intracerebral hemorrhage was similar between two periods during 8–83 days and 373–403 days after discharge [16]. On the other hand, a prospective study (n = 679) of a single ED showed that hypertensive urgencies were independently associated with increased cardiovascular events, but not with cardiovascular mortality during the follow-up period (median 4.2 years) in patients with hypertension [17]. In our study, hypertensive events were associated with both non-fatal cardiovascular events and cardiovascular mortality. However, direct comparison of the results of the present study with those of the previous studies is difficult because of the differences in the main exposures and target outcomes as well as other study characteristics including follow-up periods, population sizes, and the inclusion of general ED visitors within the study population.

A recent population-based cohort study by Masood et al. assessing ED visits with a primary diagnosis of hypertension reported a 2-year complication rate of 4.7% and a 2-year all-cause mortality rate of 3.59% in non-admitted cases [13]. In our study, the total follow-up period was longer (median 5.8 years, up to 10 years) and the cumulative incidence of MACE and cardiovascular mortality were 5.02% (4.44–5.59%) and 0.80% (0.56–1.03%), respectively. All of these findings suggest that long-term cardiovascular complications can be significant in the population.

It has generally been considered that elevated blood pressure in the ED is often caused by pain, anxiety or other ED-specific conditions; thus, it is expected to decrease spontaneously over time [18,19]. However, some studies have suggested that the association is not significant [20,21], and since many of the patients with elevated blood pressure in the ED have been re-measured and noted to be abnormal during subsequent visits [5,22,23], it has been claimed that an ED-based intervention targeting the patient group can have a significant impact. Therefore, it is possible that educating and scheduling the follow-up visit (short-term outpatient clinic or referral to a primary care provider), as recommended in current clinical policy [24], can have a beneficial long-term effect. In this study, follow-up visits after discharge significantly modified the risk of MACE during the subsequent 0–6 years, with approximately 32 to 35% reduction in the risk. This is the first evidence for the effectiveness of follow-up visits in preventing long-term complications in the population, and supports ED-based screening and education for patients with significant hypertension. However, the result of this study does not support the idea that acute antihypertensive treatment is necessary in the ED, or that the need for hospitalization is increased in case of the occurrence of a hypertensive event. Rather, our study suggests the importance of the ED as a place where long-term management of hypertension can be initiated.

Follow-up visits are of great importance, but in practice, only a small number of emergency physicians recognize it, and even fewer connections are made to the primary care physicians because of low compliance and less frequent clinic schedule appointments [25–28]. In order to improve recognition and follow-up visits, utilizing an electronic medical records system can be of beneficial by automatically referring the patients for a follow-up visit upon ED discharge. It would be also helpful to send periodic text messages reminding the relevant patients the importance of follow-up visits. Applying 24-hour ambulatory blood pressure monitoring may also be useful to determine the clinical significance of the hypertension [29]. However, according to the local treatment policy, consensus and cost-efficiency should be considered before implementing such changes. Prospective randomized studies on whether such interventions really bring about changes in long-term health should also be conducted.

This study has several limitations. First, misclassification is a potential cause of bias in a population-based study using claims records. Second, the definition for the “hypertensive event” can be arbitrary. The definition is not based on the measured blood pressure but rather on the actions of emergency physicians, including entry of diagnostic codes or administration/prescription of antihypertensive medications. However, such action-based patient identification may help to identify hypertensive population of clinical interest.

Despite the limitations, our study has several strengths. First, this is a nationwide population-based study with assessment of long-term risk of cardiovascular event up to 10 years after a non-critical ED visit with a diagnosis of hypertension or prescription of antihypertensive drugs. Another population-based study, by Masood et al. targeted patients with primary diagnosis of hypertension and observed up to 2 years using regional data. We think this study is a clinically important high-quality study [13]. However, the target population and time scope as well as the purpose of the two studies are clearly different. Second, this is the only population-based study comparing the long-term cardiovascular risk of patients with hypertensive events to those without, reporting the relative risk of cardiovascular event after hypertensive events in ED. In the study by Masood et al. there was no non-exposure group and such comparison was not possible. Third, we found the disease modifying effect of follow-up visits which had not been done previously. We think the finding can be an important evidence for supporting policies that promote ED-based hypertension screening and subsequent interventions.

## Conclusion

A hypertensive event in the ED is an independent risk factor for MACE and follow-up visit for hypertension can significantly modify the risk. Based on this evidence, we should consider implementing a nationwide intervention for risk reduction in patients with significant hypertension detected in EDs.

## Supporting information

S1 Table Operational definitions for comorbidities. (DOCX)

S2 Table Operational definitions for outcome events. (DOCX)
