# Supplementary material for: Long-term cardiovascular risk of hypertensive events in emergency department: A population-based 10-year follow-up study
Source: PLoS One. 2018 Feb 15;13(2):e0191738. doi: 10.1371/journal.pone.0191738 (PMC5813929; doi:10.1371/journal.pone.0191738)
Supplement: S1 Table — (DOCX) [file pone.0191738.s001.docx]

S1 table. Operational definitions for comorbidities

|  | international Classification of Disease (ICD)-10 criteria | Scan range (past years) | | Minimum number of events | | Additional criteria |
| --- | --- | --- | --- | --- | --- | --- |
|  |  | Admission | Non-admission | Admission | Non-admission |  |
| Hypertension | I10, I11, I12, I13, I15 | 2 | 2 | 1 | 2 |  |
| Diabetes mellitus | E10, E11, E12, E13, E14 | 2 | 2 | 1 | 2 | Should be accompanied by oral hypoglycemic agent (ATC code: A10Bx) or insulin (A10Ax) |
| Dyslipidemia | E78 | 2 | 2 | 1 | 2 |  |
| Chronic renal failure | E1021, E1121, E1221, E1321, E1421, E1022, E1122, E1222, E1322, E1422, I120, I131, I132, N18, N19 | 2 | 2 | 1 | 2 | Also include end-stage renal disease or any kidney disability registration |
| End-stage renal disease | N185, Z49, Z99, E1022, E1122, E1222, E1322, E1422 | 2 | 2 | 1 | 2 | Should be accompanied by renal replacement therapy or any patients with 1st-degree kidney disability registration |
| Ischemic heart disease | I20, I21, I22, I23, I24, I25 | 2 | 2 | 1 | 2 |  |
| Peripheral arterial disease | I70, I73 | 2 | 2 | 1 | 2 |  |
| Stroke | I60, I61, I62, I63,I64 | 2 | 2 | 1 | 2 |  |
| Heart failure | I50, I110, I255, I42, O903, I130, I132 | 2 | 2 | 1 | 2 |  |
| Advanced liver disease | I85, I864, I982, I983, K703, K704, K72, K746, K765, K766, K767, T864 | 2 | 2 | 1 | 2 |  |
| Atrial fibrillation/flutter | I48, I480, I481 | 2 | 2 | 1 | 2 |  |
| Chronic obstructive pulmonary disease | J43, J44 | 2 | 2 | 1 | 2 |  |
| Malignancy | C | 2 | 2 | 1 | 2 |  |

ATC, Anatomical Therapeutic Chemical Classification
1. Dot "." is omitted from diagnostic codes (i.e. J38.3 to J383).

2. If there are more than a code in the same hierarchy (i.e. J38 and J383), the parent codes indicate only themselves specifically, but not its children (i.e. J38x).

3. The code at the end of each of hierarchy, including those without any parent, indicate both themselves and their children (i.e. J37x for J37)
