# Supplementary material for: Long-term cardiovascular risk of hypertensive events in emergency department: A population-based 10-year follow-up study
Source: PLoS One. 2018 Feb 15;13(2):e0191738. doi: 10.1371/journal.pone.0191738 (PMC5813929; doi:10.1371/journal.pone.0191738)
Supplement: S2 Table — (DOCX) [file pone.0191738.s002.docx]

S2 table. Operational definitions for outcome events.

| Associated events | International Classification of Disease (ICD)-10 codes |
| --- | --- |
| Acute coronary syndrome | Admission with ICD-10 codes I21x, I22x, I24x, I200x (primary or the first secondary diagnosis) |
| Revascularization | Procedure code for PCI and CABG |
| Stroke | ICD-10 codes (I60x, I61x, I62x, I63x, I64x) for stroke (primary or the first secondary diagnosis) with procedure codes for brain imaging (CT/MRI) |
| Admission for heart failure | Admission with ICD-10 codes for heart failure I50x, I110x, I255x, I42x, O903x, I130x, I132x (primary or the first secondary diagnosis) |
| Pacemaker application | Procedure code for permanent pacemaker insertion or temporary transvenous/transcutaneous pacing |
| Cardiovascular death | Death certificate with diagnostic code beginning with “I” |
